# NCBP1 promotes the development of lung adenocarcinoma through up‐regulation of CUL4B

**DOI:** 10.1111/jcmm.14581

**Published:** 2019-08-26

**Authors:** Huijun Zhang, An Wang, Yulong Tan, Shaohua Wang, Qinyun Ma, Xiaofeng Chen, Zelai He

**Affiliations:** ^1^ Department of Cardiothoracic Surgery Huashan Hospital of Fudan University Shanghai China; ^2^ Department of Radiation Oncology The First Affiliated Hospital of Bengbu Medical College Bengbu China

**Keywords:** CUL4B, LUAD, NCBP1

## Abstract

Lung cancer is the most frequent cancer type and is the leading cause of tumour‐associated deaths worldwide. Nuclear cap‐binding protein 1 (NCBP1) is necessary for capped RNA processing and intracellular localization. It has been reported that silencing of NCBP1 resulted in cell growth reduction in HeLa cells. Nevertheless, its clinical significance and underlying molecular mechanisms in non–small‐cell lung cancer remain unclear. In this study, we found that NCBP1 was significantly overexpressed in lung cancer tissues and several lung cancer cell lines. Through knockdown and overexpression experiments, we showed that NCBP1 promoted lung cancer cell growth, wound healing ability, migration and epithelial‐mesenchymal transition. Mechanistically, we found that cullin 4B (CUL4B) was a downstream target gene of NCBP1 in NSCLC. NCBP1 up‐regulated CUL4B expression via interaction with nuclear cap‐binding protein 3 (NCBP3). CUL4B silencing significantly reversed NCBP1‐induced tumorigenesis in vitro. Based on these findings, we propose a model involving the NCBP1‐NCBP3‐CUL4B oncoprotein axis, providing novel insight into how CUL4B is activated and contributes to LUAD progression.

## 1 INTRODUCTION

Lung cancer is the second most common type of cancer in the West in terms of incidence, and it accounts for the majority of annual cancer deaths.^1^ Despite overall advances in lung cancer therapies, investigators continue to conduct basic science research to identify potential new therapeutic targets. Many cancer therapies, both chemotherapeutics and radiation therapies, act by inhibiting cell division and proliferation. Recently, attention has focused on post‐transcriptional regulation as a promising pathway in cancer therapeutics.^2^, ^3^ Gene expression in advanced eukaryotic cells involves multiple processes including transcription, splicing, nuclear export and translation. During these stages, specific nuclear proteins are recruited to regulate the production of nascent pre‐mRNA, resulting in the export of mature mRNA to the cytoplasm.^4^, ^5^, ^6^ Among numerous components involved in the RNA post‐transcriptional regulation mechanism, nuclear cap‐binding complex (CBC), which binds to the 5′‐end cap structure, plays an important role. Cap‐binding complex was first recognized in HeLa cells for its ability to bind to the N7‐methylated guanine (m^7^G) ‘cap structure’ of newly transcribed mRNA^7^, ^8^, ^9^ and to orchestrate downstream RNA biogenesis processes such as nuclear‐cytoplasmic transport and recruitment of translation factors in the cytoplasm.^10^ The nuclear CBC is highly conserved, from plants to humans, and consists of a heterodimer formed by nuclear cap‐binding proteins, NCPB1, NCPB2 and NCBP3, the first two of which are also referred to in the literature as cap‐binding protein 80 (CBP80) and CBP20, respectively, based on their molecular weights, whereas NCBP3 (as known as C17orf85) is a recently identified novel cap‐binding protein.^11^ NCBP2 and NCBP3 bind directly to the RNA cap, and NCBP1 stabilizes NCBP2 or NCBP3 and promotes post‐transcriptional processes. It is well documented that NCPB1 and NCPB2 participate in transcription, splicing, transcript export and translation,^12^, ^13^, ^14^ as well as the processing of histone RNA^15^ and mammalian spliceosome assembly.^16^

Proteins of the nuclear CBC may participate in functions beyond facilitating the generation and shuttling of mRNA transcripts. They may mediate responses to signals relating to cell growth and proliferation. Recently, nuclear cap‐binding proteins were implicated in abiotic stress‐responses in plants, mediating cessation of metabolic activity as a response to environmental deprivation.^17^, ^18^ One report showed that gene‐silencing of a member of the NCBP family, NCBP1, resulted in growth inhibition in HeLa cells.^11^ However, little is known about what role, if any, NCBP1 plays in stimulating growth and proliferation, such as that seen in cancer. Therefore, we conducted the present study to examine the expression of NCBP1 in lung cancer tissue and investigated the candidate target RNA, which mainly regulates tumorigenesis of lung cancer, to gain some insights into the possible mechanisms of NCBP1 involvement in lung cancer pathogenesis.

## 2 MATERIALS AND METHODS

### 2.1 Lung cancer tissue samples

Forty lung cancer tissue samples and corresponding adjacent tissues were obtained from Huashan Hospital affiliated with Fudan University. All experiments were conducted in accordance with the Declaration of Helsinki and were approved by the Ethics Committee of Huashan Hospital. All study participants gave written informed consent. To investigate the expression of NCBP1 at the mRNA level, a large cancer data set with high‐throughput sequencing data for protein‐coding genes (mRNA) was downloaded from The Cancer Genome Atlas (TCGA) (https://tcga-data.nci.nih.gov/tcga/tcgaDownload.jsp).

### 2.2 Cell lines and cultivation

Human lung cancer epithelial cell lines (HBE, A549, H1650, H838 and H1299) were purchased from the American Type Culture Collection (ATCC). All cells were maintained in RPMI‐1640 medium (Gibco BRL) containing 10% foetal bovine serum, 100 U/mL of penicillin and 100 µg/mL of streptomycin at 37°C in a humidified 5% (v/v) CO_2_ atmosphere.

### 2.3 Cell viability assays

The cell proliferation ability was assessed using a Cell Counting Kit‐8 (CCK‐8). Cells transfected with pcDNA or siRNAs were seeded into 96‐well plates at 5 × 10^3^ cells per well and were then incubated in a humidified chamber at designed intervals (0, 24, 48 and 72 hours of incubation). After 1‐2 hours of incubation with CCK8 (10 µL/well) solution, the absorbance was detected on a microplate reader at 450 nm (Bio‐Rad).

### 2.4 Quantitative real‐time PCR (RT‐qPCR)

RT‐qPCR was performed on a 7500 Fast real‐time PCR system (Applied Biosystems) using Fast SYBR Green Master mix (Applied Biosystems). Reverse transcription was performed using total‐cell RNA (TRIzol reagent, Life Technologies) and SuperScript III reverse transcriptase (Invitrogen) to quantify levels of pre‐mRNA, mRNA or eRNA. The following PCR primers were designed for this study: NCBP1 (forward primer: 5′‐GCCGGAAAAGCTGGACTTCA‐3′, reverse primer: 5′‐ATCTCCACTTCATGGGGCATC‐3′); CUL4B (forward primer: 5′‐ACTCCTCCTTTACAACCCAGG‐3′, reverse primer: 5′‐TCTTCGCATCAAACCCTACAAAC‐3′); GAPDH (forward primer: 5′‐TGTGGGCATCAATGGATTTGG‐3′, reverse primer: 5′‐ACACCATGTATTCCGGGTCAAT‐3′).

### 2.5 Western blot analysis

Total protein from cells was extracted in lysis buffer (Pierce) and quantified using the Bradford method. Then, 50 μg of protein was separated by SDS‐PAGE (10%). After transferring to polyvinylidene fluoride (PVDF) membranes (Millipore), the membranes were incubated overnight at 4°C with antibodies against CUL4B (1:1000; Cell Signaling Technology), β‐actin (1:2000, Santa Cruz Biotechnology), E‐cadherin, N‐cadherin, vimentin (1:1000, Cell Signaling Technology), NCBP1, 2 and 3 (1:1000, Abcam), and GAPDH (1:2000, Millipore). After incubation with peroxidase‐coupled antimouse IgG (Santa Cruz Biotechnology) at 37°C for 2 hours, bound proteins were visualized using ECL (Pierce) and detected using BioImaging Systems (UVP Inc). The relative protein levels were calculated based on β‐actin protein as a loading control.

### 2.6 Immunohistochemistry

Immunohistochemical (IHC) staining was performed using PowerVision™ Two‐Step Histostaining Reagent (Zhongshan Golden Bridge). For immunohistochemical staining of patient tissue samples and xenograft tumours, after deparaffinization and rehydration, tissue slides were routinely treated with 3% H_2_O_2_ at room temperature for 10 min. We used 10 mM EDTA for antigen retrieval, and then, mouse primary antibodies against human NCBP1 (1:100 for human tissues, 1:400 for mouse tissues), CUL4B (1:100 for human tissues) and Ki67 (1:400, for mouse tissues) were added to the tissues or stained with haematoxylin and eosin (HE), and incubated overnight at 4°C, followed by incubation with biotinylated secondary antibodies for 1 hour at room temperature. The expression levels of NCBP1 and CUL4B were evaluated using a scoring system based on the percentage of positive cells (0:0%‐10%; 2:10%‐30%; 4:30%‐50%; 6:50%‐80%; 8:80%‐100%).

### 2.7 Migration and wound healing assays

Transwell assays were performed to assess cell migration ability using the HTS Transwell‐24 system. For the assay, 100 μL of 5 × 10^4^ cells were seeded onto the upper chamber and the lower chamber was filled with 600 μL medium. The cells in the upper chamber without serum tended to pass through the membrane into the lower compartment with a culture medium containing 10% FBS, making it possible to estimate the ability for migration and wound healing. After incubation for 24 hours, the cells which passed through the membrane to the lower compartment were stained with Giemsa stain, fixed with 4% paraformaldehyde on the slides, and counted under a light microscope.

Cell invasion ability was determined by the wound healing assay. Cells were seeded on six‐well plates. After 24 hours, straight lines were drawn by scraping the confluent cells with a 20‐μL pipette tip and ruler. Cells floating in the medium were carefully removed, and the adherent cells were washed away with PBS three times. Culturing was continued in serum‐free medium. Following a 24‐hours incubation period, the wound healing process was monitored under a phase‐contrast microscope, the migration distance was measured, and representative images were obtained at 0 and 24 hours.

### 2.8 TUNEL assay

To evaluate the apoptotic response in tumour xenograft mouse tissues, a TUNEL assay was performed to assess in situ DNA fragmentation using a commercial kit (ApopTag Kit‐S7100, Chemicon). The incidence of apoptosis in each subgroup was quantified by counting the number of TUNEL‐positive cell nuclei under an optical microscope (Olympus) and photographed with a Cool SNAP photometric camera. The number of apoptotic cells was determined as the mean of 10 areas from each preparation.

### 2.9 RNA immunoprecipitation (RIP) assay

After the treatment of cultured lung cancer cells, an RBP immunoprecipitation kit (EMD Millipore) was used for RIP procedures according to the manufacturer's protocol. Cells were lysed in RIP lysis buffer, and then, lysates were immunoprecipitated with several antibodies (Abcam) with protein A/G magnetic beads (Sigma) overnight at 4°C. The immune complexes were immobilized with a magnet; then, bead‐bound RNA was extracted; immunoprecipitates were identified by immunoblotting of cell extracts using antibodies against NCBP1, NCBP2 and NCBP3; and standard RT‐qPCR was performed to detect CUL4B mRNA in the precipitates.

### 2.10 Plasmids and lentivirus transfection

Oligonucleotides of siRNA targeting NCBP1, CUL4B, NCBP3 and the corresponding control siRNA were obtained from Obio Technology. The sequences were presented as follows: cells were transfected at 50% confluence using Lipofectamine 2000 (Invitrogen) with a final siRNA concentration of 50 nM. NCBP1, CUL4B and NCBP3 expression plasmids were transiently transfected with X‐tremeGENE HP DNA Transfection Reagent (Invitrogen) following the manufacturer's recommendations.

Lentiviruses encoding NCBP1, NCBP1‐shRNA (sh‐NCBP1) and control shRNA (sh‐NC) were obtained from HanBio Biotechnology Co., Ltd. shRNA sequences are shown below. Cells were transfected at 50% confluence with a final lentivirus multiplicity of infection (MOI) of 20 for shRNAs. siRNA target sequences were as follows: NCBP1 (#1: 5′‐CCACAGATGATTGCTGTACTA‐3′, #2: 5′‐CAGGAACGGCACATCCTAAGA‐3′); NCBP2: 5′‐GCCAUGCGGUACAUAAAUG‐3′; NCBP3: 5′‐AAGAGCCGGTTAGATAACTTA‐3′; CUL4B: 5′‐GCCACGTACCGATACAGAAGA‐3′; shRNA target sequences were as follows: NCBP1 (#1: 5′‐ATGACTATGTATGCTGACGAA‐3′, #2: 5′‐CAGATTGAAGTTAGTCGGGAA‐3′).

### 2.11 Animal xenograft studies

Three‐ to four‐week‐old female athymic mice were purchased from Shanghai SLAC Laboratory Animal. The mice were randomly divided into three groups (5 mice/group). For each animal, H1299 cells (2 × 10^6^ cells) mixed with Matrigel (BD Biosciences) were injected into the flank. The cells had either been transfected with the negative control (shNC) or shNCBP1 plasmids. Each tumour was measured using callipers, and the tumour volume (TV) was calculated as TV = (L × W^2^)/2. To evaluate NCBP1‐related signalling in xenograft tumours, tumours were harvested after 35 days of cell transfection and were then lysed and analysed by Western blotting or qRT‐PCR and other assays. All animal care procedures accorded with institutional and international guidelines.

### 2.12 Statistical analysis

Statistical comparisons were made using a two‐tailed Student's *t* test. Quantitative data were expressed as means ± SD. Values are the result of at least three independent experiments. *P* values ≤ .05 were considered statistically significant. All analyses were performed using SPSS software.

## 3 RESULTS

### 3.1 NCBP1 is overexpressed in lung cancer tissues and cell lines

**Figure 1:**
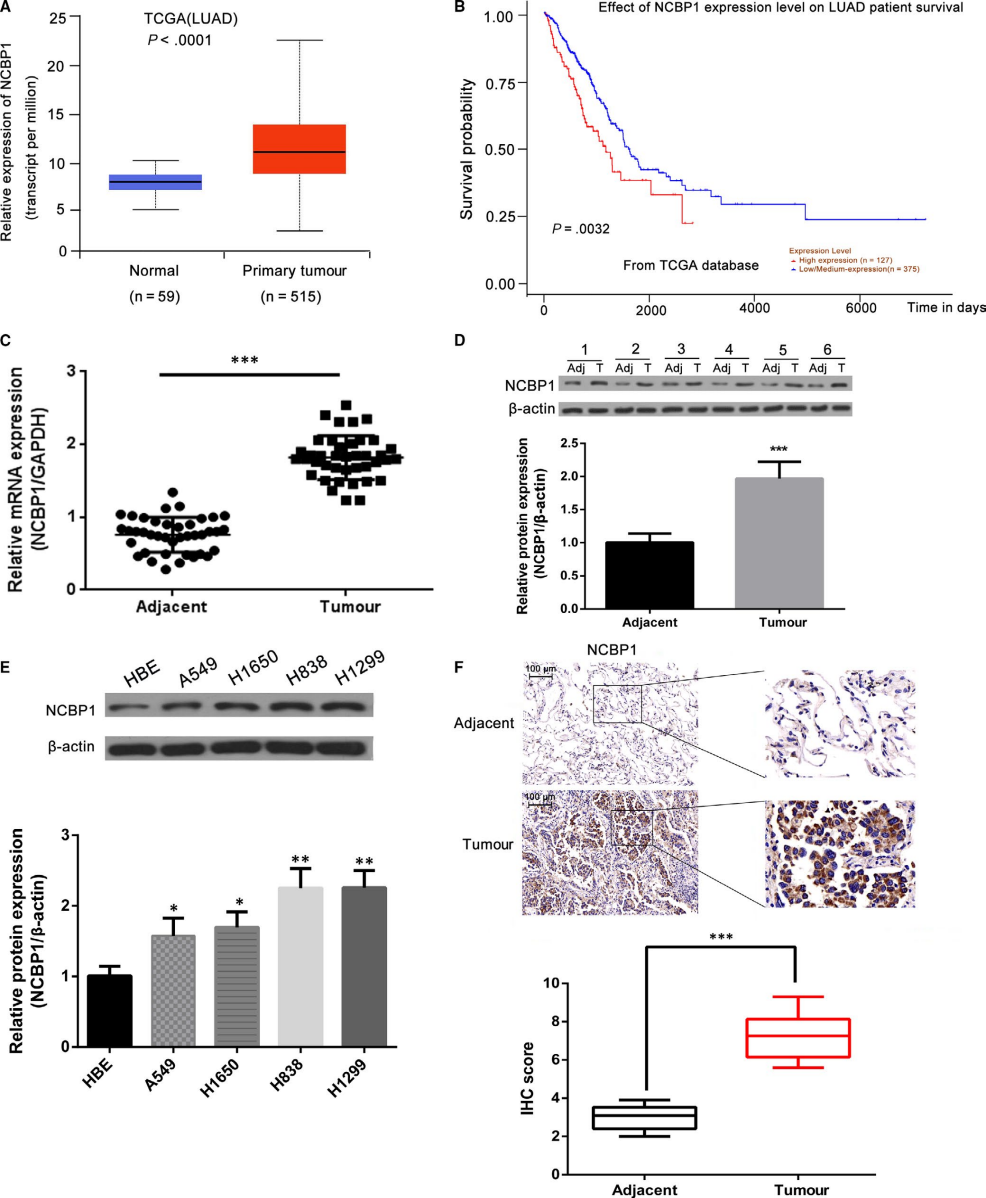
Up‐regulation of NCBP1 in lung cancer tissues and cell lines. A, mRNA expression of NCBP1 in 515 lung tumours and 59 normal lung tissues included in the TCGA database. B, Survival of patients with lung adenocarcinoma (LUAD) in the TCGA data set, predicted to have either high (red) or low (blue) NCBP1 scores. A high NCBP1 score was considered to correlate with a shortened survival time. C, D, NCBP1 expression in 40 pairs of lung cancer tissue and adjacent normal lung tissue measured by quantitative RT‐PCR and immunoblotting. Blots showed the representative results of six paired lung cancer tissues. E, NCBP1 expression in NSCLC cells and normal cells was determined by immunoblotting. F, Immunohistochemistry (IHC) evaluated the expression of NCBP1 in 40 pairs of lung cancer samples. The images shown are the representative results of one case. The staining scores of NCBP1 in lung cancer tissue were higher than those observed in the adjacent normal lung tissues. Results represent the mean ± SD; *, *P* < .05; **, *P* < .01, ***, *P* < .001. Adj, adjacent normal lung tissues; T, lung cancer tissues

The expression of NCBP1 correlates with poor survival from lung cancer. We used Kaplan‐Meier analysis to compare the predicted survival of lung adenoma (LUAD) patients (data from the TCGA database) with high expression of NCBP1 (n = 127) with that of patients with low/medium expression of NCBP1 (n = 375) (Figure 1B). LUAD patients with high expression of NCBP1 showed significantly lower survival than patients with low/medium expression (*P* = .0032). To further evaluate the role of NCBP1 in lung cancer tissues, we compared mRNA expression in 515 LUADs and 59 adjacent normal tissues from the TCGA data set (Figure 1A). NCBP1 mRNA was significantly more highly expressed in LUADs than in normal tissue (*P* < .0001). Using quantitative RT‐PCR and immunoblotting, we compared NCBP1 expression in 40 paired specimens of lung cancer tissue and adjacent normal lung tissue. NCBP1 was significantly more highly expressed in tumour tissue than in adjacent normal tissue at the mRNA level (*P* < .01) (Figure 1C), and at the protein level (*P* = .00065) (Figure 1D).

We then compared NCBP1 expression in lung cell lines and normal cells using immunoblotting (Figure 1E). We found that NCBP1 was significantly more highly expressed in cancer cells than in HBE cells (*P* < .05). NCBP1 was even more highly expressed in H838 and H1299 cells than in A549 and H1650 cells (*P* < .01). We then used immunohistochemistry (IHC) to measure NCBP1 expression in sections of patient tumour tissue samples and adjacent normal tissue (Figure 1F). Immunohistochemical scores were significantly higher in tumour tissue than in normal tissue (*P* < .001).

### 3.2 NCBP1 promotes proliferation, migration and wound healing of lung cancer cells in vitro

**Figure 2:**
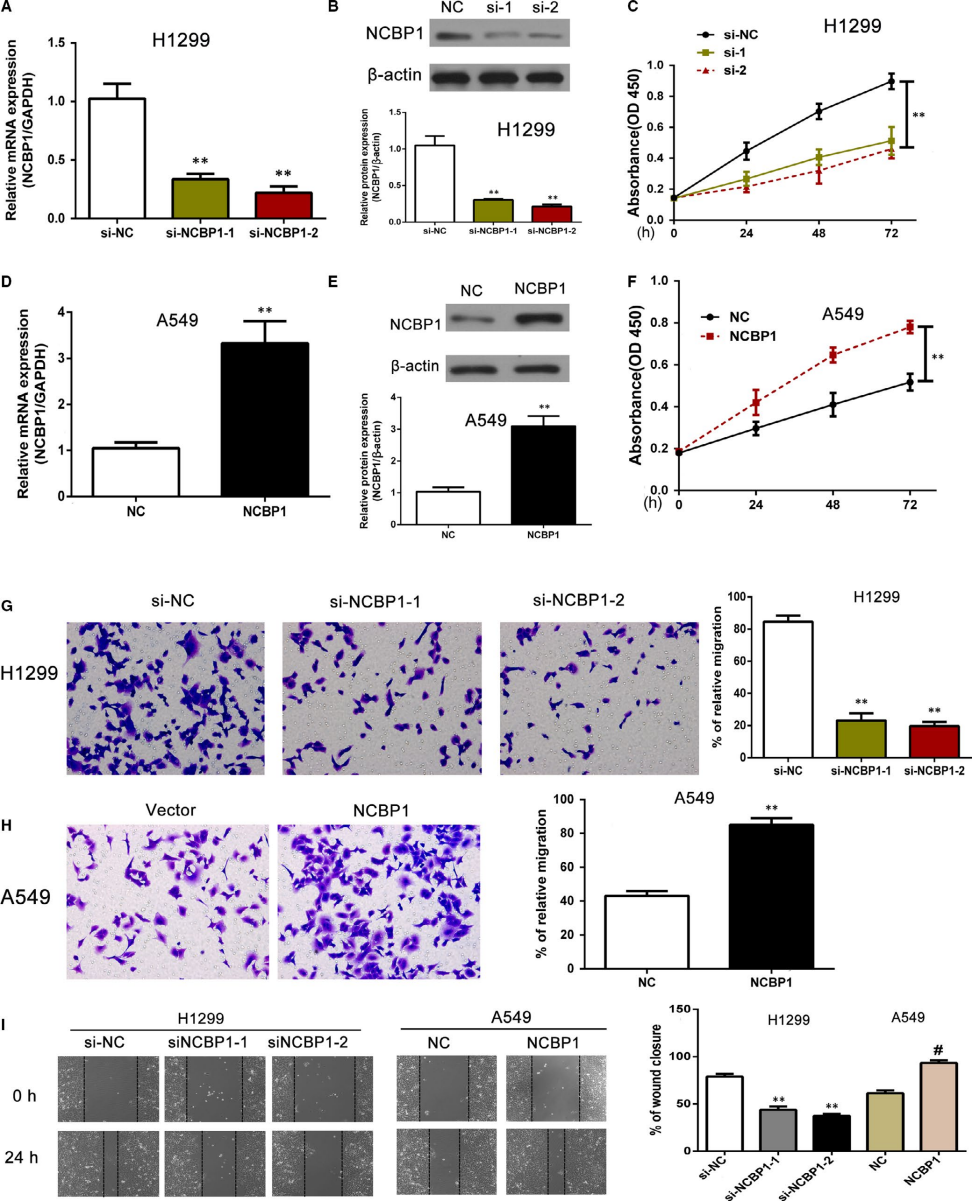
NCBP1 silencing reduced the proliferation, migration and wound healing capabilities of lung cancer cell lines, while overexpression of NCBP1 had the opposite effect. A‐C, Cell viability of H1299 cells following siNCBP1 transfection was determined by a CCK8 assay at the indicated time‐points, and knockdown efficiency of NCBP1 was confirmed by Western blotting against the indicated proteins. C‐F, A549 cells were transfected with empty (NC) or NCBP1‐expressing plasmid (NCBP1), and the NCBP1 expression levels were assessed by immunoblotting assays. The cell viability of A549 cells following NCBP1 overexpression was evaluated by a CCK8 assay at the indicated time‐points. G, H, ECMatrix gel assay to analyse the effects of siNCBP1 or NCBP1 overexpression on the migratory potential of H1299 and A549 cells. I, The migration abilities of H1299 cells (left) and A549 cells (right) with different expression levels of NCBP1 were determined by a wound healing assay at 24 h. The data are presented as the mean ± SD. Statistically significant differences were defined as follows: *, *P* < .05; **, *P* < .01; ^#^, *P* < .05

Overexpression of NCBP1 increased the proliferation, migration and wound healing of lung cancer cells, whereas the silencing of NCBP1 expression inhibited these functions. We knocked down NCBP1 expression by transfecting with siNCBP1‐1 and siNCBP1‐2 (Figure 2A,B). The knockdown efficiency of NCBP1 was confirmed by Western blotting against the indicated proteins (Figure 2B). We then performed a CCK8 assay to determine the effects of NCBP1 knockdown on H1299 cell viability (Figure 2C). We found that transfection with both siNCBP1‐1 and siNCBP1‐2 significantly inhibited cell viability at 24, 48 and 72 hours, (*P* < .01) compared with the effects of transfection with the empty plasmid (NC). We then transfected A549 cells with NC or NCBP1‐expressing plasmid (Figure 2D) and measured expression levels with immunoblots (Figure 2E). After confirming NCBP1 overexpression at the mRNA and protein levels, we measured cell viability using the CCK8 assay (Figure 2F). We found a significant increase in cell viability at 72 hours after transfection (*P* < .01), but not at 24 or 48 hours. This result suggested that NCBP1 increased cell viability in A549 cells. To measure the effects of NCBP1 overexpression and knockdown on tumour cell migration ability, we performed a migration assay (Figure 2G,H). We found that after transfection of H1299 cells with both siNCBP1‐1 and siNCBP1‐2, there was significantly less migration than in H1299 cells transfected with empty plasmid (*P* < .01). By contrast, there was significantly greater migration in A549 cells transfected with NCBP1‐containing plasmid than with empty plasmid (*P* < .01).

We performed a wound assay to study the effects of NCBP1 on tumour cell invasiveness using a wound healing assay (Figure 2I). We found that migration into artificially induced wounds was significantly higher in H1299 (*P* < .01) and A549 (*P* < .05) cells transfected with NCBP1 plasmid than with empty plasmid. Conversely, NCBP1 knockdown with siNCBP1‐1 and siNCBP1‐2 significantly inhibited migration of H1299 cells into wounds.

### 3.3 CUL4B is up‐regulated in lung cancer cells and is moderately correlated with NCBP1 expression

**Figure 3:**
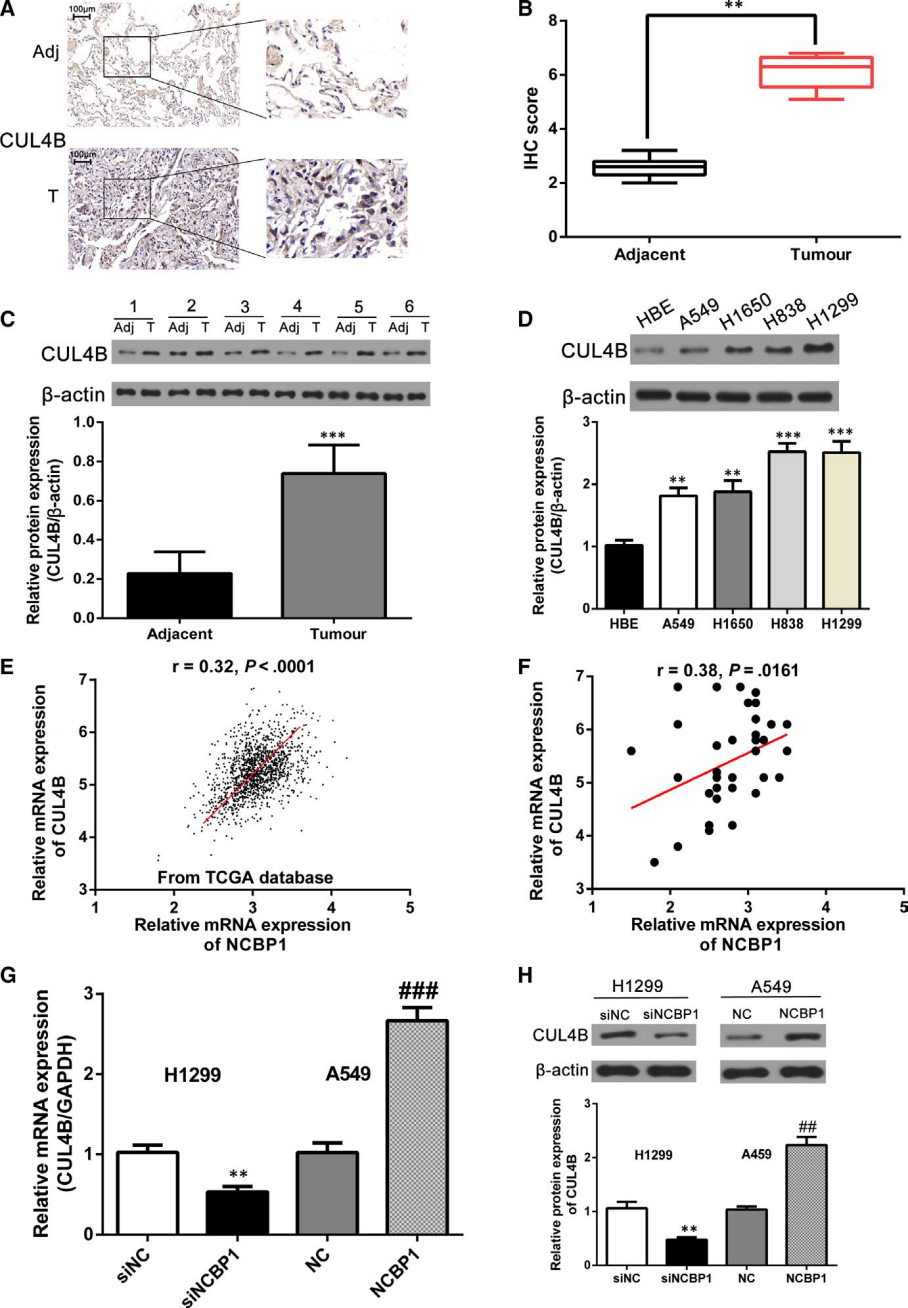
Identification of CUL4B as a downstream target of NCBP1. A, B, Immunohistochemistry (IHC) evaluated the expression of CUL4B in 40 pairs of lung cancer samples. Images shown are the representative results of one case. The staining scores of CUL4B in lung cancer tissue were higher than those observed in the adjacent normal lung tissues. C, D, CUL4B expression in 40 pairs of lung cancer tissue and NSCLC cells was detected by immunoblotting. E, CUL4B expression was significantly correlated, albeit moderately, with the expression of NCBP1 (*r* = .32; *P* < .0001) in lung cancer tissue (data from the TCGA database). F, Verification of the relationship between CUL4B and NCBP1 in 40 pairs of lung cancer samples (*r* = .38; *P* = .0161). G, H, Quantification of CUL4B protein levels was performed by Western blotting after transfection with siNCBP1 in H1299 cells and NCBP1 overexpression vectors in A549 cells. Data represent the mean ± SD, **, *P* < .01; ***, *P* < .001; ^##^, *P* < .01

To investigate the role of cullin 4B (CUL4B) and its relationship with NCBP1 in lung cancer cells, we first measured CUL4B levels in tumours and adjacent tissues from lung cancer patients (Figure 3A‐D). We found that CUL4B was significantly more highly expressed in tumour tissue than in adjacent tissue at both the protein level (Figure 3A‐C) and the protein level in cancer cell lines (Figure 3D). Using the TCGA data set, we found that expression of CUL4B mRNA was significantly correlated, albeit moderately, with that of NCBP1 in lung cancer tissue (*r* = .32; *P* < .0001) (Figure 3E). We verified this relationship in 40 pairs of lung cancer tissues from patients (Figure 3F). Again, CUL4B mRNA expression correlated with NCBP1 mRNA expression (*r* = .38; *P* = .0161). To further explore the relationship between NCBP1 and CUL4B expression, we measured CUL4B protein expression in lung cancer lines with and without overexpression or knockdown of NCBP1 (Figure 3G,H). We found that CUL4B protein expression was significantly lower in H1299 cells transfected with siNCBP1 (*P* < .01) compared with empty plasmid. By contrast, CUL4B protein expression was significantly higher in A549 cells transfected with NCBP1 plasmid compared with empty plasmid (*P* < .01).

### 3.4 CUL4B is a key mediator of the phenotype induced by NCBP1 activation

**Figure 4:**
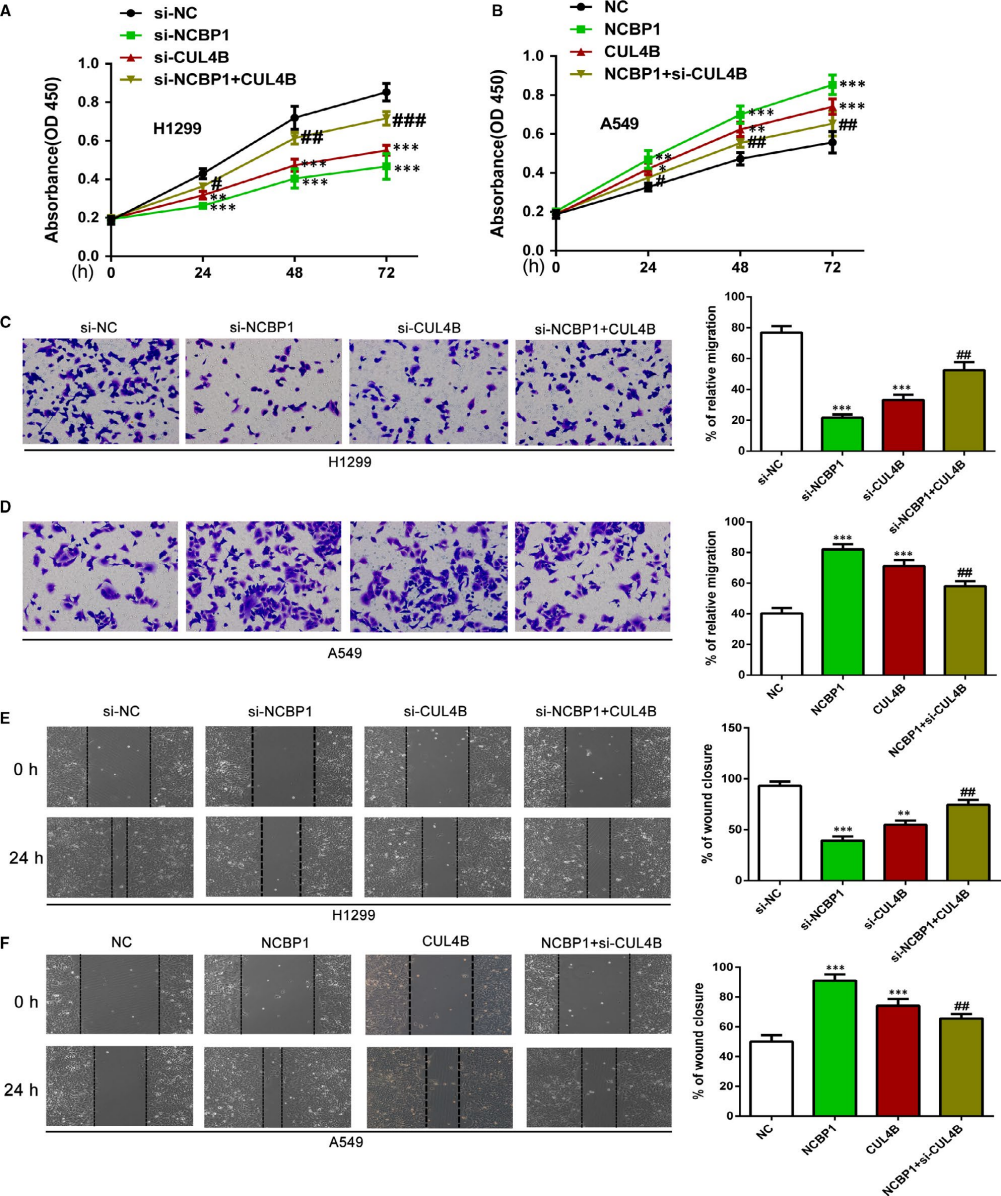
NCBP1 regulates cell proliferation, migration and wound healing ability, and this is partly dependent on CUL4B. A, The viability of H1299 cells following siNCBP1 or siCUL4B transfection and cotransfection with siNCBP1 and CUL4B was determined by a CCK8 assay at the indicated time‐points. The growth inhibition of siNCBP1 was reversed following CUL4B overexpression. B, Cell viability of A549 cells following NCBP1 or CUL4B overexpression and cotransfection with NCBP1 and siCUL4B was determined by a CCK8 assay at the indicated time‐points. The facilitation of growth by NCBP1 was inhibited following CUL4B knockdown. C, D, ECMatrix gel assay to analyse the effects of siNCBP1 or siCUL4B, CUL4B overexpression together with NCBP1 knockdown, NCBP1 or CUL4B overexpression and NCBP1 overexpression with CUL4B knockdown, on the migratory potential of H1299 and A549 cells. E, F, The migration abilities of H1299 cells (E) and A549 cells (F) with different expression levels of NCBP1 and CUL4B were determined by a wound healing assay at 24 h. The results represent the mean ± SD; **, *P* < .01; ***, *P* < .001; ^#^, *P* < .05; ^##^, *P* < .01

To examine the role of CUL4B as a mediator of the action of NCBP1 in lung cancer, we measured the viability of lung cancer cell lines with various combinations of plasmid transfections. We found that knockdown of NCBP1 or CUL4B significantly inhibited cell proliferation in H1299 cells (Figure 4A) (*P* < .01), but cotransfection with CUL4B plasmid partially reversed this effect (*P* < .001). The transfection of A549 cells with NCBP1 or CUL4B plasmid significantly enhanced viability (*P* < .001), and cotransfection with siCUL4B partially blocked this effect (Figure 4B) (*P* < .01). We then performed parallel experiments, looking at the role CUL4B in mediating the effects of NCBP1 on cancer cell migration. We found that migration of H1299 cells was significantly inhibited after transfection with siNCBP1 or siCUL4B (*P* < .001) and that this effect was partially reversed by cotransfection with CUL4B plasmid (*P* < .01) (Figure 4C). By contrast, migration of A549 cells was significantly enhanced by transfection with NCBP1 or CUL4B plasmid (*P* < .001) and this effect was partially blocked by cotransfection with siCUL4B plasmid (*P* < .01) (Figure 4D). We then performed wound healing assays to explore the role of CUL4B in mediating the effects of NCBP1. We found that transfection of H1299 cells (Figure 4E) or A549 cells (Figure 4F) had varying effects on wound healing. Silencing of NCBP1 or CUL4B significantly inhibited wound healing (*P* < .001), and cotransfection with CUL4B plasmid partially reversed this effect (*P* < .01).

### 3.5 NCBP1 modulates the expression of epithelial‐mesenchymal transition‐associated proteins through CUL4B

**Figure 5:**
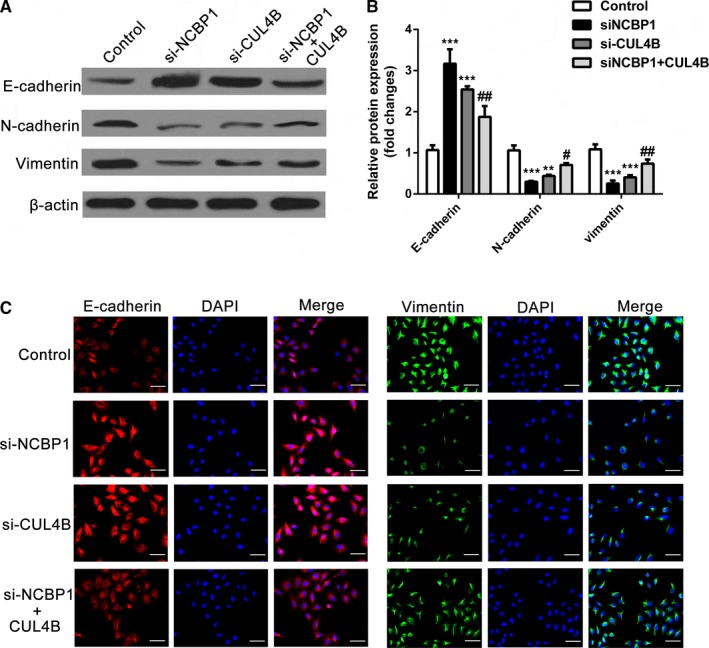
NCBP1 modulates EMT‐associated proteins through CLU4B. A, B, Representative Western blot analysis and relative protein levels of CUL4B, E‐cadherin (E‐cad), N‐cadherin (N‐cad) and vimentin in H1299 cells with NCBP1 knockdown and cotransfection with siNCBP1 or siCUL4B and CUL4B plasmid together with NCBP1 knockdown. C, Cell immunofluorescence of EMT‐associated proteins in the above group. Scale bar = 20 μm, red indicates E‐cadherin, and green indicates vimentin

To examine the role of NCBP1 and CUL4B in epithelial‐mesenchymal transition (EMT), we measured the levels of CUL4B and various epithelial and mesenchymal markers at the protein level (Figure 5A‐C) in H1299 cells. Loss of E‐cadherin expression correlates with EMT, cancer progression and metastasis. We found that silencing NCBP1 or CUL4B expression in H1299 cells significantly increased E‐cadherin protein expression (*P* < .01) and that cotransfection with CUL4B plasmid partially reversed this effect (*P* < .05). Mesenchymal markers such as N‐cadherin and vimentin are also markers of EMT and cancer progression. We found that silencing NCBP1 or CUL4B expression in H1299 cells significantly inhibited N‐cadherin and vimentin expression (*P* < .01) and cotransfection with CUL4B plasmid partially reversed this effect (*P* < .05) (Figure 5A‐C).

### 3.6 NCBP1 together with NCBP3 promotes CUL4B mRNA expression

**Figure 6:**
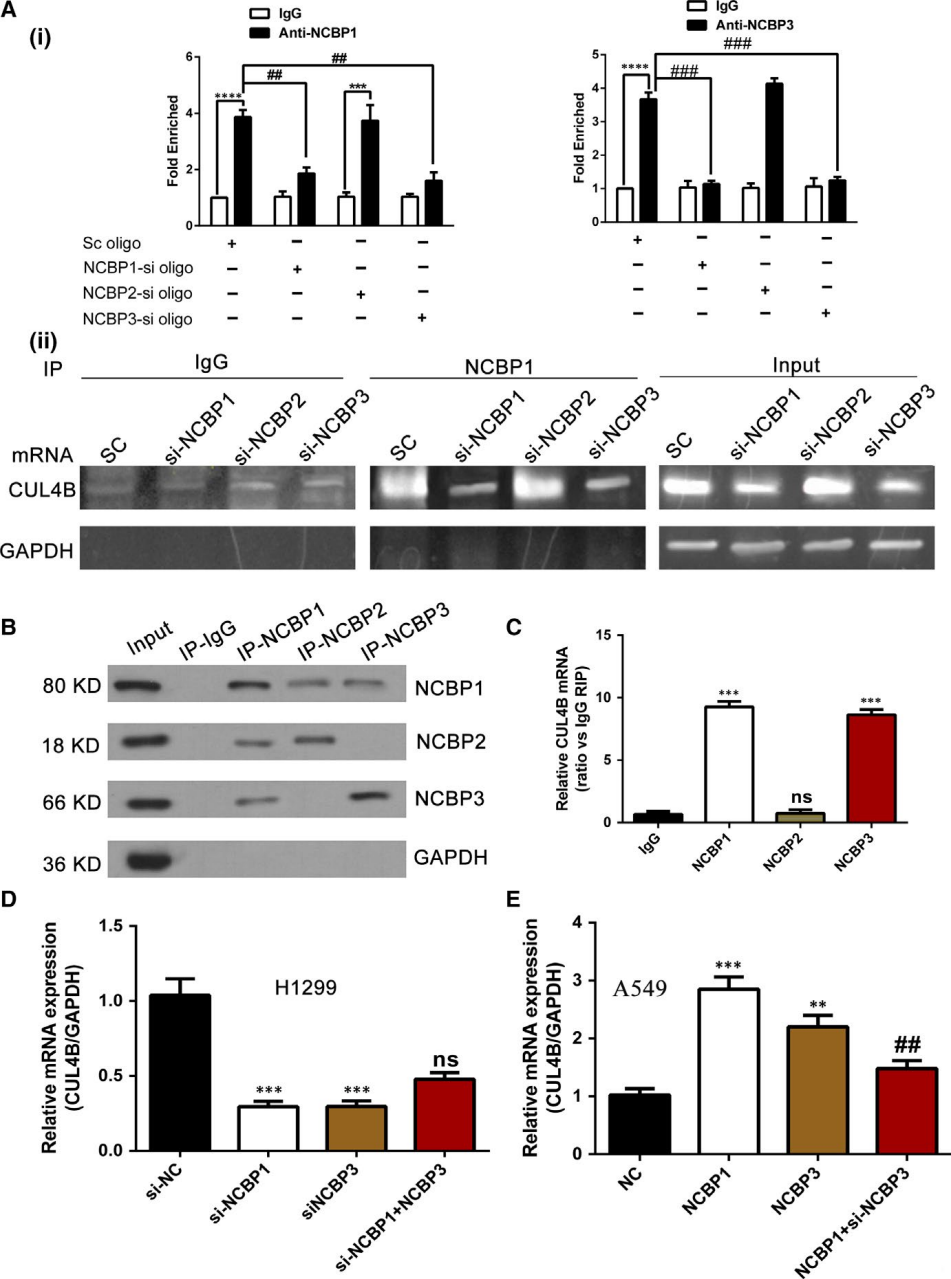
NCBP1 together with NCBP3 regulates CUL4B mRNA expression. A, RNA immunoprecipitation assay. Cellular extracts of H1299 cells transfected with the sc, NCBP1‐si, NCBP2‐si or NCBP3‐si were immunoprecipitated with NCBP1 or NCBP3 antibodies, and the target mRNA in the immunoprecipitation material was evaluated through quantitative RT‐PCR (i) and semi‐quantitative RT‐PCR (ii) using primers against CUL4B mRNA. GAPDH was analysed is a negative control. B, C, RIP was performed using lysates from H1299 cells with NCBP1, NCBP2 and NCBP3 antibodies, and the precipitated materials were analysed by Western blot analysis using the indicated antibodies. Enrichment of CUL4B mRNA was measured using quantitative RT‐qPCR. D, H1299 cells were transfected with siNC, siNCBP1, siNCBP3 and siNCBP1 + NCBP3 plasmids to detect CUL4B mRNA expression by quantitative RT‐PCR. NCBP3 overexpression did not reverse the inhibition of CUL4B expression by siNCBP1. E, A549 cells transfected with NCBP1 plasmid, NCBP3 plasmid or NCBP1 plasmid together with siNCBP3 or the empty plasmid as a control. NCBP3 knockdown reduced CUL4B promotion of NCBP1 overexpression. Data are reported as the mean ± SD (n = 3); **, *P* < .01; ***, *P* < .001; ^#^, *P* < .05; ^##^, *P* < .01

Alternative components of the nuclear CBC were recently described, referred to here as NCBP1 and NCBP3. To further explore the regulation of CUL4B mRNA stability by NCBP1 and NCBP3, we first detected whether NCBP1 and NCBP3 could form an protein‐RNA complex together with CUL4B mRNA by carrying out RNA immunoprecipitation (RIP) assays on lysates of H1299 cells with NCBP1, NCBP2 or NCBP3 knockdown. Furthermore, CUL4B mRNA expression in the immunoprecipitated complexes was examined by quantitative (Figure 6A‐i) and semi‐quantitative (Figure 6A‐ii) RT‐PCR. We found that anti‐NCBP1 antibody pulled down CUL4B mRNA in the NCBP1 or NCBP3 knockdown groups to a significantly lesser extent than in the NCBP2 knockdown and control groups. We also performed these pull‐down assays using antibodies against NCBP1, NCBP2 and NCBP3 (Figure 6B). We found that anti‐NCBP1 antibodies pulled down NCBP2 and NCBP3, anti‐NCBP2 antibodies pulled down NCBP1 but not NCBP3, and anti‐NCBP3 antibodies pulled down NCBP1 but not NCBP2. We also performed standard RT‐qPCR to detect CUL4B in the immunoprecipitates (Figure 6C) and found that anti‐NCBP1 and anti‐NCBP3 pulled down significantly more CUL4B mRNA than the control or anti‐NCBP2 (*P* < .001).

We then transfected H1299 cells with empty plasmid, siNCBP1, siNCBP3 or siNCBP1 + NCBP3 plasmid to detect CUL4B mRNA expression by quantitative RT‐PCR (Figure 6D). We found that NCBP3 overexpression did not reverse the inhibition of CUL4B expression induced by siNCBP1 (*P* < .0001). When we transfected A549 cells with NCBP1 plasmid, NCBP3 plasmid or NCBP1 plasmid together with siNCBP3, and found that NCBP3 silencing reduced the CUL4B promotion of NCBP1 overexpression (Figure 6E) (*P* < .01).

### 3.7 Inhibition of NCBP1 reduced the development of lung cancer in vivo

**Figure 7:**
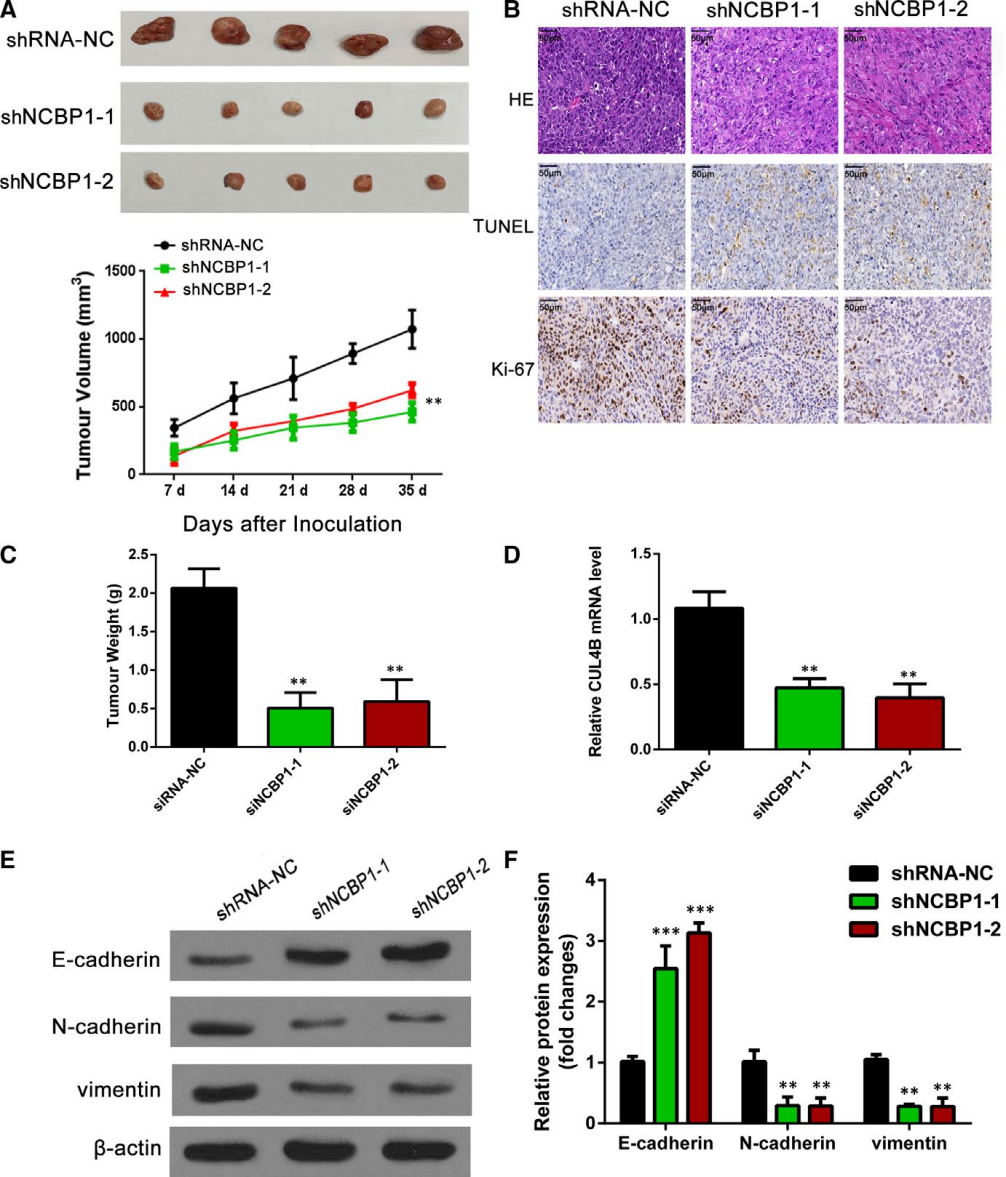
NCBP1 promoted tumour growth in a mouse lung cancer xenograft model. A, C, Nude mice were inoculated subcutaneously with H1299 cells and were divided into three treatment groups: control siRNA‐NC, siRNA NCBP1‐1 and siRNA NCBP1‐2. Subsequently, the xenograft tumour size was monitored every 7 days (volume = width^2^ × length × 1/2). Points represent the mean tumour volumes of three independent experiments (n = 5). After 35 days, the xenograft tumours were excised from the nude mice, and their weights are presented in panel c. B, Cancer cell morphology, proliferation (Ki‐67) and apoptosis (TUNEL) in the xenograft tumours were examined by H&E, TUNEL and immunohistochemical staining, and data were quantified with Image ProPlus (IPP) software (Media Cybernetics). D, RT‐PCR of CUL4B mRNA expression in lung cancer xenografts following NCBP1 knockdown. E, F, The levels of the major EMT key proteins were detected in the xenograft tumours by immunoblotting assays. The data are presented as the means ± SD. *, *P* < .05; **, *P* < .01, ***, *P* < .001

To test the effects of NCBP1 in vivo, we established a mouse lung cancer xenograft model (Figure 7). We subcutaneously injected nude mice with H1299 cells that had been previously transfected with shRNA‐NC (control), shRNA NCBP1‐1 or shRNA NCBP1‐2. After 35 days, xenograft tumour volumes (Figure 7A) and weights (Figure 7C) were significantly lower in NCBP1‐silenced tumours than in control tumours (*P* < .01). NCBP1‐silenced tumours showed fewer cancer‐specific morphological features than control tumours; Ki‐67 staining suggested there was a lower degree of proliferation in NCBP1‐silenced tumours; and a TUNEL assay suggested that there were greater levels of apoptosis in NCBP1‐silenced tumours than in the controls (Figure 7B). Tumours in which NCBP1 was silenced showed significantly lower levels of CUL4B mRNA expression (Figure 7D) (*P* < .01).

Epithelial–mesenchymal transition was also substantially inhibited in NCBP1‐silenced xenografts, the levels of E‐cadherin were significantly greater (*P* < .001), and the levels of N‐cadherin and vimentin were significantly lower (*P* < .01) compared with the controls (Figure 7E,F).

## 4 DISCUSSION

Many studies have reported the role of cap‐dependent protein translation in oncogenesis, and translation initiation factors have long been recognized as potential sources of dysregulation during the oncogenic process.^19^, ^20^, ^21^ However, the role of nuclear cap‐binding protein 1 in the development and progression of cancer is not well understood. For the first time, we found that NCBP1 expression was significantly higher in lung cancer tissue than in non‐cancerous lung tissue. Furthermore, elevated NCBP1 expression correlated with a poor prognosis in patients with lung adenocarcinoma, based on data from the TCGA database, but unfortunately, we lacked clinical survival data. NCBP1 expression varied in established cancer cell lines, with the highest expression seen in H838 and H1299 cells. We used two lung cancer cell lines with NCBP1 knockdown and overexpression to systematically address the role of NCBP1 in the proliferation and migration of lung cancer cells. Down‐regulation of NCBP1 decreased the proliferation and migration of H1299 cells, whereas the opposite effect was observed with overexpression of NCBP1. These data suggested that NCBP1 may play at least a promotional role in the proliferation and migration of lung cancer cells.

Cullin 4B (CUL4B) is a member of the cullin 4 subfamily of genes that includes CUL4A, with which it shares an almost identical amino acid sequence. These molecules act as scaffolding proteins for modular cullin RING ligase 4 (CRL4) complexes that promote the ubiquitination of a variety of substrates.^22^, ^23^, ^24^ Although accumulating research indicates that CUL4B expression is significantly elevated in various cancers including lung cancer,^25^, ^26^, ^27^, ^28^ the molecular mechanism of CUL4B up‐regulation remains unclear. In this study, we demonstrated that NCBP1 knockdown decreased CUL4B expression, whereas NCBP1 overexpression improved CUL4B expression. Given that CUL4B is an important ubiquitination‐related molecule, along with the fact that NCBP1 promotes the proliferation, migration and wound healing ability of lung cancer cells, we attempted to elucidate the underlying regulatory mechanism of CUL4B mediated by NCBP1.

In this research, we found that CUL4B expression correlated with that of NCBP1 in lung cancer tissue and in cancer cell lines, and cotransfection with CUL4B plasmids partially reversed the effects of NCBP1 silencing on lung cancer cell line proliferation, migration and wound invasion. Our in vivo study showed that silencing of NCBP1 reduced tumour size as well as affecting the morphological and biochemical characteristics of the tumours and simultaneously decreasing CUL4B expression.

Taken together, these data suggest that NCBP1 mediates, at least in part, the proliferation, migration and invasion characteristics, as well as EMT, in lung cancer cells, and that CUL4B may be a downstream mediator of the effects of NCBP1.

Evidence is accumulating that post‐transcriptional regulation mechanisms might play an important role in determination of the gene expression level. It is apparent that NCBP1 has extensive activities that depend on its interaction partners. For example, PGC‐1α promotes transcription of the large gene through binding to NCBP1 in HEK293T cells.^29^ A previous study demonstrated that NCBP1 guides mRNA from the nucleus into the cytoplasm by directly binding with NCBP2 or NCBP3 in HeLa cells.^11^ After transcription initiation, a heterodimer of NCBP1/‐2 or NCBP1/‐3 immediately forms combined with the cap structure at the 5′‐end of the pre‐mRNA. It is noticeable that NCBP1 binds to mRNA indirectly because it lacks a canonical cap recognition domain; hence, it stabilizes NCBP2 or NCBP3 and serves as an adaptor for other factors to the capped RNA.^30^

In this study, we verified (Figure 6) that either NCBP1 or NCBP3 interacts with CUL4B mRNA and silences NCBP3, decreasing NCBP1‐mediated CUL4B mRNA expression; however, NCBP overexpression cannot reverse the lower expression of CUL4B induced by NCBP1 knockdown. It has been reported that depletion of NCBP1 deregulated expression of numerous genes and reduced the cell proliferation rate.^31^ Consistent with this research, our results indicated that NCBP1 is vital for maintaining CUL4B mRNA expression, but its function partly depends on NCBP3. This suggested that NCBP3 might serve as a bridge between RNA‐binding proteins during mRNA biogenesis, acting upstream at mRNA export. RNA immunoprecipitation experiments indicated the specificity of RNA types bound by NCBP2 and NCBP3. As previously reported, NCBP2 showed particularly high binding with snRNA, lincRNA and asRNA, and by contrast, NCBP3 was associated with mRNA, but did not bind to snRNA, and bound comparably less asRNA and lincRNA.^8^, ^11^

Our findings revealed an oncoprotein‐RNA axis, providing novel insight into how CUL4B is activated and contributes to lung adenocarcinoma progression. The limitation of this study was that we did not verify whether NCBP1 together with NCBP3 combined to the 5′‐end cap structure of CUL4B pre‐mRNA, regulating CUL4B mRNA export from the nucleus. The exact mechanism involved in NCBP1/‐3‐mediated metabolism of CUL4B mRNA and exploring this axis as an avenue for the development of new cancer therapeutics will be the subject of our further studies.

## 5 CONCLUSION

Our study reveals the potential role of NCBP biogenesis factors as putative players in lung cancer cell proliferation, migration and EMT that may contribute to tumour development.

## CONFLICT OF INTEREST

The authors declare that there are no conflicts of interest.

## AUTHOR CONTRIBUTIONS

XFC and ZLH designed and guided the project; HJZ carried out the experiments and wrote the manuscript; AW and YLT performed statistical analysis and modified the manuscript; and SHW and QYM collected clinical samples and assisted HJZ in animal experiments. All authors approved the final manuscript.

## Data Availability

All other data are available upon request.
